# Putative Effects of Lead on the Endocannabinoid System: A Literature Review and Summary

**DOI:** 10.3390/ijms26188994

**Published:** 2025-09-16

**Authors:** Gersham J. Rainone, Phillip Mitchell Johansen, Peter Pressman, Andrew Wallace Hayes

**Affiliations:** 1 Department of Neurosurgery and Brain Repair, University of South Florida, Tampa, FL 33620, USA; johansenp@usf.edu; 2 Institute of Medicine & Department of Sociology, University of Maine, Orono, ME 04469, USA; drpressvm2@gmail.com; 3 Center for Environmental/Occupational Risk Analysis & Management, College of Public Health, University of South Florida, Tampa, FL 33620, USA; awallacehayes@comcast.net

**Keywords:** lead, cannabinoid, receptors, biochemistry, toxicology

## Abstract

Lead is a naturally occurring metal found in numerous compounds used in everyday life. Toxicity from lead is a well-known public health problem. Its effects are implicated in multiple tissues, encompassing the gastrointestinal, renal, cardiovascular, and neurological systems. Endocannabinoid receptors are involved in each of these systems, but the effects of lead on the receptors themselves are not well elucidated. In the neurological system, lead has varying interactions with neurotransmitters and downstream regulators implicated in neuronal transmissions influenced by endocannabinoid receptor function. Lead’s effect is likely indirect on endocannabinoid receptor function; however, its influence on neuronal function is likely inhibitory to the receptor’s functioning. Lead has also been implicated in oxidative stress states, which would influence endocannabinoid receptors’ function. The literature clearly supports lead having a negative impact on the overall function of endocannabinoid receptors, setting the stage for pathological states related to diminished neurosynaptic function and, in embryology, altered neuronal development, especially of the neural tube.

## 1. Introduction

Heavy metals have been extensively studied, with known pathophysiological effects. Lead toxicity involves complex interactions between environmental exposure, biological absorption, and cellular disruption. It affects multiple organ systems, with children being particularly vulnerable to its neurotoxic effects. Early recognition, reduction in exposure, and appropriate management are essential for minimizing the long-term impacts of lead on human health. The central nervous system is particularly sensitive to alterations in metabolism and synaptic function that heavy metals can influence [1,2,3,4].

Lead is used in the modern world due to its softness, malleability, ductility, poor conductivity, and resistance to corrosion [1,5]. Before 1980, lead was a ubiquitous material that permeated infrastructure, industry, and daily lives worldwide. Its applications—from plumbing and paint to gasoline and electronics—were as varied as they were consequential. While its practical uses helped shape modern civilization, the discovery of its toxic effects marked a turning point, prompting one of the most significant public health initiatives of the 20th century. The legacy of lead, both beneficial and hazardous, continues to inform policy, science, and industry to this day [6,7].

Understanding the sources, behavior, and potential risks of lead in food and soil is essential for protecting human health and ensuring food safety. Lead is a pervasive environmental contaminant with serious health implications, particularly for children. Its complex interactions within soil and subsequent plant uptake directly impact food safety and human health. Lead in contaminated soil tends to accumulate in the top few inches. It primarily exists in solid forms and binds tightly to fine clay and organic matter particles. While lead is not highly soluble in water, its bioavailability to plants is influenced by soil pH, organic matter content, and certain minerals. Plants generally do not absorb large quantities of lead. The primary concern regarding lead in plants grown in contaminated soil is the adherence of lead-contaminated soil dust or particles to the plant’s surface. However, some plants can absorb lead, particularly leafy greens and root vegetables. Lead enters the food chain through the direct consumption of plants grown in contaminated soil or by lead dust settling on food crops. 

In the United States, while adding lead to food is illegal, lead has been found in certain foods and spices, particularly imported varieties. In 2024, several cinnamon products, including applesauce pouches and ground cinnamon, were recalled in the USA due to elevated lead levels. Other examples include imported candy containing chili powder, tamarind, and certain spices. Improper drying, storage, or processing can also contribute to lead contamination. Taking appropriate precautions and implementing effective management strategies can reduce lead exposure, thereby safeguarding public health.

The greatest vulnerability to lead toxicity involves the central nervous system, especially during embryonic development. Lead exposure adversely affects the central nervous system by crossing the blood–brain barrier and disrupting neural processes, with children being especially vulnerable to cognitive impairments, behavioral changes, and delayed neurological development, while adults may experience peripheral neuropathy, cognitive decline, and mood disorders. These effects result from mechanisms including neurotransmitter interference, impaired synaptic pruning, oxidative stress, and calcium signaling disruption, often causing permanent neurological damage and highlighting the importance of reducing environmental lead exposure [6,8,9].

The endocannabinoid receptors, CB1 and CB2, are crucial in early central nervous system development and function and play a role in neurotransmission in the fully developed adult nervous system [10]. The direct effect of lead on the endocannabinoid receptors has not been well studied; however, there are numerous indirect effects of lead on the endocannabinoid pathway cascade that can lead to alterations in neurotransmission and synaptic function, in turn leading to pathologic states in the central nervous system [10,11].

## 2. The Endocannabinoid System

### 2.1. Endocannabinoid Receptors

The endocannabinoid system (ECS), a complex and widely distributed signaling network, is a vital physiological network that regulates numerous bodily functions by maintaining internal balance and responding to environmental changes [12,13,14,15,16,17]. It comprises endogenous cannabinoids, receptors, and enzymes that work together to influence health and disease. The system includes endocannabinoids such as anandamide and 2-arachidonoylglycerol (2-AG), cannabinoid receptors (CB1 and CB2), and enzymes such as fatty acid amide hydrolase (FAAH) and monoacylglycerol lipase (MAGL) that synthesize and degrade these signaling molecules on demand. CB1 receptors are mainly in the central nervous system, affecting movement, memory, and pain, while CB2 receptors are found in the peripheral nervous system, particularly immune cells, modulating inflammation and immunity [14,15,16,17,18,19]. CB1 and CB2 are G-protein-coupled receptors (GPCR) that couple with and interact primarily with inhibitory GPCRs. The ECS regulates homeostasis, pain, memory, immune response, sleep, neuroprotection, mood, appetite, and metabolism by modulating neurotransmitter release and acting as retrograde messengers in neural communication. Dysfunctions in the ECS are linked to chronic pain, neurological, autoimmune, mental health, metabolic, and sleep disorders. The ECS has been linked to numerous body systems beyond the central nervous system including the reproductive system and gastrointestinal system [20]. Therapeutic strategies include drugs targeting ECS signaling and medical cannabis, with ongoing research into safety, dose–response patterns, tolerance, personalized medicine, and novel ECS components [12,13,14,15,16,17,18,19,21,22,23,24,25,26,27].

### 2.2. Retrograde Signaling

Endocannabinoids play a significant role in retrograde signaling, in which activation of the receptor at the synapse by an endocannabinoid can influence synaptic release of neurotransmitters and other essential chemical messengers [21,24,25,27,28]. They also play a larger overall role in neuronal excitability via modulation of ion channels. Endocannabinoids’ most crucial role in the central nervous system is during development [10,11]. During gestation, endocannabinoid receptors are abundant in the cortex and the hippocampus, including the amygdala, the cerebellum, and the basal ganglia [14,15,26,29,30]. They are also found in axonal tracts that traverse the cortex to the spinal cord, such as the corticospinal and corticothalamic tracts [15]. Interruption in their signaling or alterations in the composition or expression of endocannabinoid receptors in these tracks can lead to deviant paths of these critical neuronal elements. The prefrontal cortex elements are also rich in endocannabinoid content and receptor expression, which is responsible for numerous post-natal development aspects. Endocannabinoid receptor signaling has also been implicated in cilium and sonic hedgehog functions in developing embryos, which are critical for early neuronal development [8]. These pathways, especially retrograde signaling, have been implicated in developing and closing the neural tube during gestation. Failure of closure of the neural tube leads to a classification of disorders known as neural tube defects, which are responsible for significant morbidity and mortality as development continues. Neural tube defects that can result from this, which can be classified as open or closed, include anencephaly, spina bifida, myelomeningocele, craniorachischisis, lip myelomeningocele, tethered cord, Chiari malformation, and spinal dysraphism.

## 3. The Metabolism of Lead

### 3.1. Lead Toxicokinetics

The toxicokinetics of Pb in humans have been extensively studied, and several models have been published that simulate the absorption, complex distribution and elimination of Pb from blood, soft tissues, and bone. Inorganic Pb can be absorbed following inhalation, oral, and dermal exposure, but the last route is much less efficient than the former [3]. Inorganic Pb in small, submicron-sized particles is readily absorbed through the respiratory tract. In contrast, larger particles can be cleared from the respiratory tract by mucociliary clearance toward the oropharynx and swallowed, leading to gastrointestinal absorption of Pb. The fraction of ingested Pb absorbed from the gastrointestinal tract depends on age, diet, nutrition, and the physiological characteristics of Pb in the medium ingested [3]. Children absorb 40–50% of an oral dose of water-soluble Pb compared to 3–10% for adults. Gastrointestinal absorption of inorganic Pb occurs primarily in the duodenum via saturable mechanisms. The presence of food in the gastrointestinal tract decreases the absorption of Pb. Pb absorption in children can be affected by nutritional iron status and the literature suggests that iron deficiency may result in higher absorption of Pb [3].

### 3.2. Metabolic Processing of Lead

The distribution of Pb in the body is route independent. In adults, approximately 94% of the total body burden of Pb is in the bones compared to approximately 73% in children. Pb in blood is primarily in red blood cells. Conditions such as pregnancy, lactation, menopause, and osteoporosis increase bone resorption and blood Pb concentration. Pb can be transferred from mother to the fetus and infants via maternal milk [3]. Metabolism of inorganic Pb consists of the formation of complexes with various protein and nonprotein ligands. Organic Pb compounds are metabolized in the liver by oxidative dealkylation by P-450 enzymes. Pb is excreted primarily in urine and feces regardless of the route of exposure. Minor routes of excretion include sweat, saliva, hair, nails, breast milk, and seminal fluid. Elimination of Pb is multiphasic, reflecting pools of Pb in the body that have varying retention times. The apparent elimination half-time in blood varies with age and exposure history and ranges from 1 week to 2 years. Elimination of Pb from bone occurs with an apparent half-time of 1–2 decades [3,4].

## 4. Effect of Lead on the Central Nervous System

**Figure 1:**
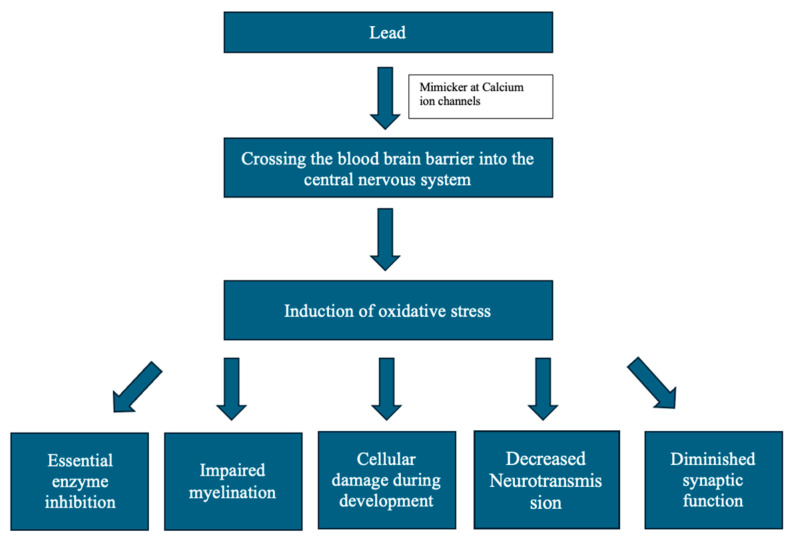
Effects of lead on the cellular and molecular functioning of the central nervous system.

Lead is a persistent environmental neurotoxin that poses serious risks to the central nervous system (CNS), especially in children whose developing brains are highly vulnerable. Exposure occurs through inhalation, ingestion, or skin contact with sources like lead-based paint, contaminated soil, and water. The toxic effects of lead are multifaceted and can cause lasting cognitive, behavioral, and neurological damage [1,3,4], which are demonstrated in Figure 1. Lead disrupts neurological processes by mimicking calcium, inducing oxidative stress, altering neurotransmitter systems, inhibiting essential enzymes, and impairing myelination, collectively damaging neuronal function and communication. Children absorb more lead and have immature blood–brain barriers, making them especially susceptible during critical brain development. Increased susceptibility and absorption therefore lead to greater effects on neuronal and glial tissues, giving way to more critical and function-threatening pathologies. The physical and cognitive symptoms that are commonly seen in lead poisoning can be explained at the molecular level in each of the brain areas that lead typically affects.

### 4.1. Anatomic Effects of Lead

Important anatomical areas commonly affected by lead poisoning include the prefrontal cortex, hippocampus, and cerebellum. Impaired prefrontal cortex development can lead to difficulty with executive functioning and higher-order processing, such as memory, planning, decision making, impulse control, emotional regulation, and social behavior. Impaired development of the prefrontal cortex in children who are affected by lead poisoning predisposes them to developmental delay, severe learning disabilities, and impaired abilities to interact socially. This can lead to poor school and test performance and difficulty with interacting with their peers, leading to further derangements in their independent functioning, commonly leading to dependence on others for their care. Altered function of the hippocampus has implications in memory consolidation and retrieval, emotional regulation, spatial navigation, and learning. This can lead to immense difficulties for children exposed to lead, due to diminished spatial awareness, poor spatial memory and pattern recognition, as well as poor overall learning ability, test performance, and ability to carry out simple tasks. Abnormal development of the cerebellum can cause hindrances in balance and coordination, gait coordination, posture, and voluntary muscle activity. This is often how symptoms can present in children exposed to lead, with difficulty walking or moving, and falls due to the body’s inability to coordinate and recognize its place in time and space. These anatomic landmarks of the brain implicated in lead poisoning are essential to functioning in activities of everyday life. Thus, exposure to lead in children can result in reduced IQ, attention deficits, learning disabilities, and behavioral problems such as impulsivity and aggression.

### 4.2. Detection of Treatment of Lead Exposure

While adults are less sensitive, chronic lead exposure can cause peripheral neuropathy, cognitive decline, language and motor delays, and increased risks of mood disorders and neurodegenerative diseases. The elevated risk of mood disorders and neurodegenerative diseases is likely secondary to the detrimental effects that lead has on the metabolism and synaptic transmission that neuronal and glial cells are reliant upon for proper functioning. Blood lead levels are used to detect exposure, though bone lead measurements better reflect long-term burden. Treatment includes removal from exposure, chelation therapy, and supportive care, but prevention remains the most effective strategy to reduce lead’s neurotoxic impact [3]. Common chelating therapies for lead poisoning in adults and children are calcium disodium ethylenediaminetetraacetic acid, succimer, and dimercaprol (with succimer more commonly being used in children). These agents are able to neutralize heavy metals by donating electrons, which are used to form covalent bonds with the metal ion. These covalent bonds help to form a ring-like structure, in turn holding the metal in place, allowing for sequestration of the heavy metal, filtration by the renal system, and excretion in the urine.

### 4.3. Molecular Effects of Lead

Lead passes through the blood-brain-barrier via calcium ion channels at the endothelial junctions of the barrier, and into neuronal tissue by the Calcium-ATPase [1,6]. It affects neuronal and glial cells, indirectly altering metabolism and impairing the synthesis of essential building blocks. Lead decreases the production of neurotransmitters, namely glutamate, and alters the expression of proteins involved in ATPase function and the formation and organization of synapses and ion channels [1,2,5,6,8]. Lead has also been shown to interfere with myelin production and myelin sheath formation, which is essential for the propagation of neuronal signals throughout neural networks [1,9]. In adults, lead poisoning is associated with cerebral atrophy and detrimental effects in the prefrontal cortex. Lead has been shown to have a more detrimental effect in children, even at lower doses. Similarly to adults, who on MRI have shown significant atrophy and alterations in the prefrontal cortex after lead exposure, children exposed to lead have been linked to numerous neuropsychiatric conditions and have decreased cognitive performance. In children and neonates, lead has also been shown to be detrimental to the development of neuronal cells in the hippocampus, a brain region crucial for learning and memory. It affects neurotransmission and synapse function by interfering with glutamate metabolism, and has been shown to bind selectively to N-methyl-D-aspartate (NMDA) receptors and induce apoptosis [1,8,9].

Lead has been shown to have incredibly detrimental effects on numerous anatomical areas of the brain situated in different lobes and locations. Lead’s toxic effects on a molecular basis are translated into alterations in the overall function of the neuronal cells and glial tissue in the anatomic areas described above, leading to diminished efficiency and effectiveness of function in these critical anatomic areas. The anatomic locations of lead’s effects relate directly to the symptoms outwardly seen in both children and adults affected by exposure.

## 5. Lead and the Endocannabinoid System

Lead (Pb) is a pervasive environmental pollutant with a long history of human exposure and well-documented toxic effects, particularly on the nervous system. While much is known about the impact of lead on cognitive and developmental processes, emerging evidence suggests that lead exposure may also interact with the endocannabinoid system (ECS)—a critical neuro-modulatory network involved in maintaining homeostasis within the brain and peripheral organs. There is much overlap seen in the downstream effects of lead on particular anatomy in the central nervous system—including the prefrontal cortex, hippocampus, and cerebellum—whose development is predicated on the proper functioning and interaction of the endocannabinoid system with neurotransmitters and other essential neural elements.

### 5.1. Molecular Overlap

There have not been any conclusive studies in human cell lines. However, some data support interactions of lead with neural elements and substrates that are important to and involved in endocannabinoid signaling. Lead exposure, therefore, has the potential to pose significant neurotoxic risks, including disrupting the endocannabinoid system (ECS), which plays a crucial role in brain development, immune response, and synaptic function. Understanding this interaction is vital for developing preventive and therapeutic strategies. Lead interferes with the synthesis, release, and degradation of endocannabinoids, altering levels of key molecules like AEA and 2-AG, and affecting enzymes such as FAAH and MAGL, impairing neural communication and plasticity. Lead exposure changes the expression and function of cannabinoid receptors CB1 and CB2, reducing CB1 density in brain regions important for cognition and upregulating CB2 in immune cells, contributing to neuroinflammation and cognitive deficits. Lead increases reactive oxygen species, causing oxidative damage, while ECS normally offers protective effects; persistent lead exposure overwhelms this system, leading to neuronal damage and ECS dysregulation. Disrupted ECS during critical brain development stages can result in learning disabilities, ADHD, mood disorders, and increased risk for neurodegenerative diseases, alongside behavioral changes like impulsivity and anxiety [3,4,14,15,18,31,32].

### 5.2. Neonatal Development and Signaling Pathways

Starting with neonatal development, endocannabinoid receptors have been implicated in the development of critical structures of the cortex, including areas responsible for memory and learning, transmission of long tract fibers, and executive functioning [6]. These areas of the brain are affected by lead poisoning in utero, drawing a potential connection between the two in these anatomical structures. Endocannabinoid receptors, particularly by retrograde signaling, are involved in sonic hedgehog signaling pathways responsible for the neural tube’s development and closure [9,11,31]. Lead has also been implicated in neural tube defects via inhibition of ligands of the sonic hedgehog pathway, raising the question of whether there is an interaction between lead and endocannabinoid receptors during development in neonates exposed to lead. In neonates, children, and adults alike, lead exposure has been linked to diminished functioning and abnormal development of the prefrontal cortex. Studies have reported that the prefrontal cortex and the hippocampus are enriched in endocannabinoid receptor content, and interestingly enough, these are two areas of the cortex that are particularly affected by lead poisoning, as demonstrated by MRI findings in multiple studies. This begs the question of whether there is a direct interaction between lead and endocannabinoid receptors, as they are implicated in similar pathologies concerning anatomy [10,11,18,23,24,25,26,29,31,32].

Involvement of both lead and the endocannabinoid system in sonic hedgehog signaling pathways also raises the question of their roles in pathologic states where sonic hedgehog is overexpressed, such as basal cell carcinoma, chordoma, soft tissue sarcomas, and various other types of malignancies, in which children exposed to lead are at higher risk of developing. The endocannabinoid system’s effect on sonic hedgehog signaling is generally considered inhibitory to prevent overexpression. Lead’s role as an inhibitor of the adequate functioning of the endocannabinoid system, therefore, could logically lead to overexpression of sonic hedgehog signaling, thus implicating it in malignant processes, which has yet to be explored in detail.

Previous studies have demonstrated that lead is detrimental to retrograde signaling pathways, one of the most useful and primary functions of endocannabinoid receptors in the central nervous system [10,15,18,27]. Lead has been shown to dampen the interaction between endocannabinoids and calcium channels via downregulation of GPCR’s, which are commonly associated with endocannabinoid receptors and in alterations of lipid metabolism, which, in turn, would lead to a decrease in precursor substrates responsible for producing the major endocannabinoids: anandamide and 2- arachidonoylglycerol [19,27]. Some data have suggested that lead can be inhibitory at the synapse, in turn affecting the interaction of substrates and receptors. This response would impair the release of substrates such as glutamine, dopamine, and serotonin from synapses in the cortex and brainstem. Synapses in the central nervous system are rich in endocannabinoid receptors; thus, their signaling pathways and effectiveness may be blunted by the presence of lead in an instance of lead poisoning.

### 5.3. Oxidative Stress

**Figure 2:**
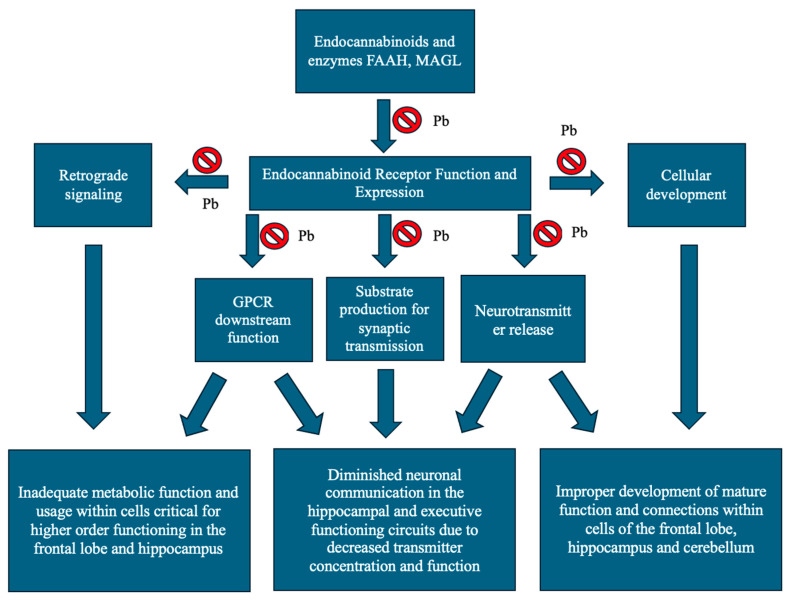
Lead’s potential impact on the endocannabinoid system and its downstream neurological consequences in neurotransmission and function in the frontal lobe, hippocampus and cerebellum. Substrate production for synaptic transmission and neurotransmitter release effects of lead inhibit functioning of neuronal pathways in the function of memory circuits and executive functioning in the hippocampus and frontal lobes. Lead’s effects on retrograde signaling have impact on numerous CNS functions, as does GPCR function and cellular development.

Another likely point of interaction between lead and endocannabinoid receptors would be through oxidative stress. Like most heavy metals, lead can induce oxidative stress on the nervous system’s cells. Oxidative stress leads to free radical formation and alterations in protein structure, metabolism, and cell signaling. Retrograde signaling and the interaction between endocannabinoid receptors and GPCR’s may be altered in oxidative stress, as neuronal cells shift to protect themselves, leading to potential downregulation of endocannabinoid receptors and their downstream effects. Although these effects have not been well studied in human cell lines, they pose important questions about the interactions of this heavy metal with a receptor system that we are growing to better understand, outlined in Figure 2.

## 6. Methods

The literature was queried in the following manner. PubMed and Google Scholar databases were queried for results with the following search terms: lead, poisoning, endocannabinoid, and central nervous system. Members of our investigative team analyzed results to determine their relevance to the topic at hand. Manuscripts detailing lead’s interaction with the endocannabinoid system, lead’s interaction with the central nervous system, and detailed accounts of the receptor signaling pathways for the endocannabinoid system were investigated further by our team to extract information for our manuscript. Members of our team then processed the information to fit into the over-arching categories described above in the manuscript.

## 7. Conclusions and Future Research Aims

Although there are no definitive links or rigorously focused studies of the interaction between lead and the endocannabinoid system, significant overlaps as suggested warrant further investigation. Lead has been shown to decrease retrograde signaling, diminish the function of the prefrontal cortex and hippocampus, impair the function of proteins and signaling, leading to neural tube defects, and alter metabolism at the synapse—all of which can be directly related to endocannabinoid function. Further studies should investigate the role of cell expression of endocannabinoid receptors in the presence of lead. This work should investigate the production of downstream components of endocannabinoid signaling in the same light. Again, while direct human studies linking lead and ECS biomarkers are sparse, it is tempting to speculate that lead may cause epigenetic changes via altered DNA methylation or histone acetylation that may in turn affect ECS gene regulation. This interaction might explain long-term changes in ECS function following early-life lead exposure. Future experiments and investigations could include quantifying endocannabinoid receptor expression and downstream protein levels in neuronal cell lines containing lead versus a control without lead exposure. Other experimental ideas include measuring metabolic output of endocannabinoid induced cells in the hippocampal, frontal lobe and cerebellar regions of rats or mice exposed to lead versus controls without exposure. Other prospective areas for future research might include human studies measuring lead levels vs. ECS biomarkers, and interventions using ECS-targeting drugs to reverse or buffer lead-induced damage.

## Abbreviations

The following abbreviations are used in this manuscript:

ECS	The endocannabinoid system
Pb	Lead
2-AG	2-arachidonoylglycerol
FAAH	Fatty acid amide hydrolase
MAGL	Monoacylglycerol lipase
GPCR	G-protein-coupled receptors
CNS	Central nervous system
NMDA	N-methyl-D-aspartate
